# Comparing the predictive roles of intrinsic and extraneous cognitive load on learning performance: the mediating effect of IVR self-efficacy in warehouse training

**DOI:** 10.3389/fpsyg.2026.1838395

**Published:** 2026-06-24

**Authors:** Yen-Ting Chiang, Jon-Chao Hong, Yu-Ting Tu

**Affiliations:** 1 Department of Industrial Education at National Taiwan Normal University, Taipei, Taiwan; 2 Institute for Research Excellence in Learning Sciences, National Taiwan Normal University, Taipei, Taiwan

**Keywords:** extraneous cognitive load (ECL), immersive virtual reality (IVR), learning outcome, operational cognitive load (OCL), self-efficacy, warehouse safety training

## Abstract

**Introduction:**

IVR-Warehouse is an immersive virtual reality (IVR) educational game designed to train senior high technical students in warehouse safety, addressing the high incidence of occupational accidents in Taiwan’s warehouse environments. The system enables learners to practice hazard identification and safety responses in a realistic, risk-free environment, thereby overcoming the logistical and safety limitations of real-world training. This study examined the relationship between task-specific IVR self-efficacy, extraneous cognitive load (ECL), VR operational cognitive load (OCL), and learning outcome.

**Methods:**

Structural equation modeling was applied to data collected from 167 participants. The results showed that task-specific IVR self-efficacy was negatively associated with both ECL and OCL, and that both forms of cognitive burden were negatively associated with learning outcome.

**Results:**

Notably, OCL showed a stronger predictive effect than ECL, suggesting that operational demands, such as interface access, controller use, attention maintenance, and action sequencing, posed a greater challenge for learners than general system- or environment-related distractions. These findings highlight the importance of reducing operational burden and supporting learners’ task-specific confidence to maximize the effectiveness of IVR-based warehouse safety training.

**Conclusion:**

These findings underscore the importance of managing content difficulty to maximize the effectiveness of IVR-based safety training.

## 1 Introduction

Safety training is essential for maintaining operational efficiency and reducing both the likelihood and severity of workplace hazards (Makransky and Petersen, 2021). In Taiwan, recent data from the Occupational Safety and Health Administration, Ministry of Labor, Taiwan (2024) indicate that common occupational accidents in the warehousing industry include falls, entanglement, cuts, and collision-related injuries, with collision-related injuries showing particularly high occurrence across industries (Occupational Safety and Health Administration, Ministry of Labor, Taiwan, n.d.). The frequency of disabling injuries in this sector is 5.49 per 1,000 workers, and the total injury index is 1.12, reflecting a 12% higher injury risk compared with the national average over the past three years.

Given these safety concerns, immersive virtual reality (IVR) offers a promising approach to safety training by allowing learners to rehearse procedures in realistic yet safe environments (Guo et al., 2024). Prior research has shown that IVR-based interactive scenarios can improve safety behaviors by providing situated, hands-on practice (Hong et al., 2023; Mahammedi et al., 2025). As warehouse operations become increasingly complex, effective and adaptable training methods are urgently needed (Al-Hamad et al., 2025). IVR has particular potential because it can be customized to reflect specific occupational hazards, support safety culture development, and align training with particular learning settings and workplace contexts (Al-Hamad et al., 2025; Lesňák et al., 2025). Despite this pedagogical potential, IVR-based safety training programs remain limited for Taiwanese students in warehouse-specific vocational education contexts. To address this gap, the present study developed IVR-Warehouse, an immersive training game designed to enhance safety awareness and hazard response skills among senior high technical students.

Beyond its practical application, IVR also provides a valuable context for examining perception, embodiment, and learning processes. Metzinger (2018) argues that immersive environments can give rise to a “transparent self-model,” through which users experience embodied presence and mentally inhabit the virtual space (Lehrman, 2025). This view corresponds to embodied cognition theory, which suggests that physical interaction with the environment contributes to cognitive processing (Shapiro and Spaulding, 2021). Likewise, social cognitive theory emphasizes self-efficacy as a critical mechanism for supporting learners’ cognitive engagement and learning experiences in IVR contexts (Ateş and Kölemen, 2025). In this study, self-efficacy is conceptualized as task-specific IVR self-efficacy, referring to learners’ perceived capability to operate the IVR-Warehouse system and complete the required safety training tasks. Higher levels of task-specific IVR self-efficacy may strengthen learners’ psychological readiness, reduce perceived cognitive burden, and support learning outcome (Al-Kfairy et al., 2025). However, such findings were obtained in contexts different from warehouse safety training and may not be directly transferable to senior high technical students learning hazard identification and safety-response skills. Therefore, the present study examined how task-specific IVR self-efficacy shapes cognitive load and learning outcomes within an IVR-based warehouse safety simulation.

### 1.1 Purpose of the study

IVR can recreate hazardous scenarios that would be dangerous or impractical to stage in real life, providing learners with a convincing sense of “being there” (McIntosh, 2022) while fostering hazard-identification and safety-response skills (Hayes and Fowler, 2025; Kuo and Cheng, 2025). In this study, these skills refer specifically to learners’ ability to recognize warehouse hazards, respond appropriately to unsafe situations, and complete safety-related actions in the simulated warehouse environment. To fully benefit from such immersive experiences, however, learners’ self-beliefs play an important role. Bandura’s Personal Self-Efficacy (SEP) refers to “people’s beliefs about their capabilities to produce designated levels of performance” (Bandura, 1994, p. 71; Bandura, 1997). SEP is widely regarded as one of the determinants of performance because individuals with higher SEP tend to show greater effort, persistence, and confidence when completing challenging tasks. In the present study, SEP was operationalized as task-specific IVR self-efficacy, referring to learners’ perceived capability to operate the IVR-Warehouse system and complete the required warehouse safety training tasks.

To explain how cognitive burden affects learning, researchers often draw on Cognitive Load Theory (Paas et al., 2003; Sweller et al., 1998). In IVR-based training, learners must not only process instructional content but also operate controllers, access interface commands, maintain attention, and follow task sequences. Therefore, this study focused on two forms of cognitive burden: extraneous cognitive load (ECL), which arises from unnecessary system- or environment-related distractions, and VR operational cognitive load (OCL), which reflects the perceived burden associated with operating the IVR system and completing action sequences. Excessive ECL and OCL may reduce learners’ available cognitive resources and hinder learning outcome, particularly among novice IVR users.

Accordingly, this study examined how task-specific IVR self-efficacy relates to ECL, OCL, and learning outcomes in IVR-based warehouse safety training. Specifically, it explored whether ECL and OCL differentially predict learning outcome and whether task-specific IVR self-efficacy indirectly influences learning outcomes through its effects on these two forms of cognitive burden.

### 1.2 Research question

Cognitive Load Theory (CLT) distinguishes intrinsic, extraneous, and germane cognitive load (Plass and Kalyuga, 2019). Intrinsic cognitive load refers to the inherent complexity of learning materials, whereas extraneous cognitive load (ECL) arises from unnecessary demands caused by instructional design, interface features, or inefficient information presentation (Sweller et al., 1998; Skulmowski, 2023). However, in immersive virtual reality (IVR), learners must simultaneously process safety content, operate controllers, navigate spatial interfaces, and complete interactive task sequences, making cognitive-load classification more complex. In this study, the construct originally labeled as ICL was relabeled as VR operational cognitive load (OCL) because the retained items reflected attention maintenance, interface accessibility, controller difficulty, and action sequencing rather than the inherent complexity of the learning material. Accordingly, this study examined the predictive roles of ECL and OCL in learning outcome within an IVR-based warehouse safety training context.

RQ: To what extent do ECL and OCL predict learning outcome in IVR-based warehouse safety training, and does OCL exert a stronger negative effect on learning outcome than ECL?

### 1.3 Hypotheses

Within the CLT framework, an important distinction is made between mental load the demands a task imposes on the learner and mental effort the cognitive resources allocated to handle those demands (Paas and van Merriënboer, 1994). This distinction invites consideration of how learners perceive and respond to task difficulty, whether through their expectancy of achieving a learning goal, their personal self-efficacy (SEP), or their perceived ease of learning (Scheiter, 2025). For example, Du et al. (2025) found that cognitive load mediated the relationship between self-efficacy and learning achievement, suggesting that learners with higher self-efficacy may achieve better performance partly because they experience lower cognitive burden during learning. In IVR-based warehouse safety training, learners must process safety-related information while simultaneously operating controllers, navigating the virtual environment, and completing task-based actions. Therefore, learners with higher task-specific SEP may feel more capable of managing both the learning task and the operational demands of the IVR system. In this study, SEP was operationalized as task-specific IVR self-efficacy, referring to learners’ perceived capability to operate the IVR-Warehouse system and complete the required hazard-identification and safety-response tasks. Accordingly, students with higher task-specific IVR self-efficacy were expected to experience lower extraneous cognitive load (ECL) and VR operational cognitive load (OCL).

*H1*: Task-specific IVR self-efficacy is negatively related to ECL.

*H2*: Task-specific IVR self-efficacy is negatively related to OCL.

Cognitive load can influence how learners process information, form schemas, and retain knowledge (Paas and van Merriënboer, 2020). In IVR environments, ECL may arise from system-related distractions, response delays, disorientation, or object manipulation difficulties, whereas OCL reflects the operational burden associated with attention maintenance, interface access, controller use, and action sequencing. Both forms of cognitive burden may reduce learners’ available cognitive resources and hinder learning outcome, especially when students are unfamiliar with the IVR environment. Thus, the following hypotheses were proposed:

*H3*: ECL is negatively related to learning outcomes.

*H4*: OCL is negatively related to learning outcomes.

Self-efficacy also plays a vital role in technology adoption, engagement, and performance. Learners with higher task-specific IVR self-efficacy may be better able to manage operational challenges and reduce unnecessary cognitive burden, thereby supporting learning outcomes. Previous research has also demonstrated that cognitive load can mediate the relationship between self-efficacy and achievement (Du et al., 2025). Therefore, the following mediation hypothesis was proposed:

*H5*: Task-specific IVR self-efficacy is positively related to learning outcomes, mediated by ECL and OCL.

## 2 Method and material

### 2.1 IVR-Warehouse

**Figure 1:**
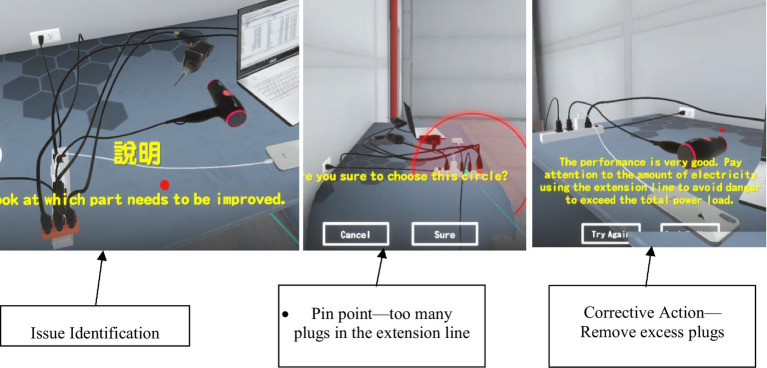
VR-Warehouse electrical safety training.

**Figure 2:**
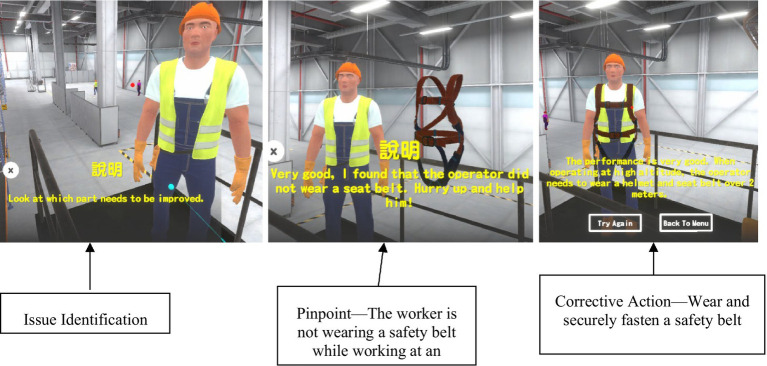
VR-Warehouse high-altitude operation and safety.

**Figure 3:**
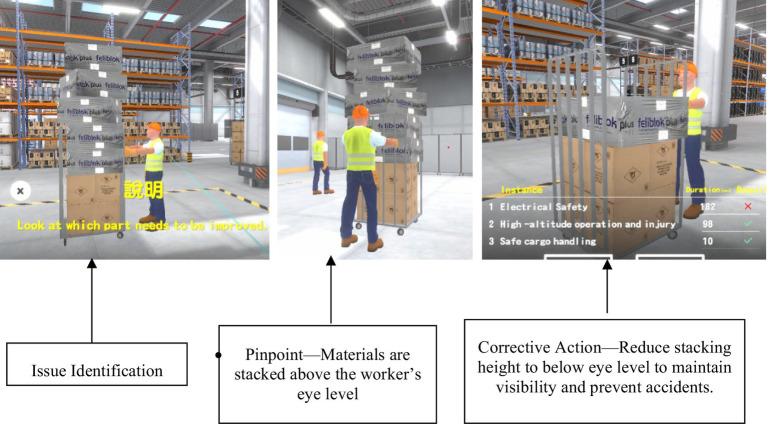
VR-Warehouse safe-cargo handling.

IVR-Warehouse, formally named *IVR Warehouse Risk Awareness*, is an immersive virtual reality training system developed by the research team in 2022 to simulate common workplace hazards in Taiwanese warehouse environments. The system was developed using Unity 2023.2 and deployed on an Acer OJO 500 VR headset. Participants interacted with the virtual environment using two handheld controllers, which allowed them to point, select, confirm responses, and manipulate virtual objects. The system aims to improve learners’ hazard awareness and safety-response skills through scaffolded tasks. Users are required to identify unsafe conditions, select the corresponding hazards, and complete corrective actions before progressing to the next stage. The platform includes individual training and simulation modes. The simulation mode integrates multiple tasks into a continuous learning experience, whereas the assessment function provides real-time feedback on accuracy, response speed, and decision-making. Figures 1–3 show the three modules used in this study: Working at Heights, Electrical Safety, and Safe Cargo Handling (Table 1).

**Table 1:** Ten training categories in IVR-Warehouse.

1 Storage environment	2 Electrical safety	3 High-altitude operation and safety	4 Injury	5 Safe-cargo handling
6 Mobile ladder operation safety	7 Electrical spark	8 Forklift safety operations	9 Obstructed or slippery passage	10 Item storage and stacking

### 2.2 Experimental procedure

**Figure 4:**
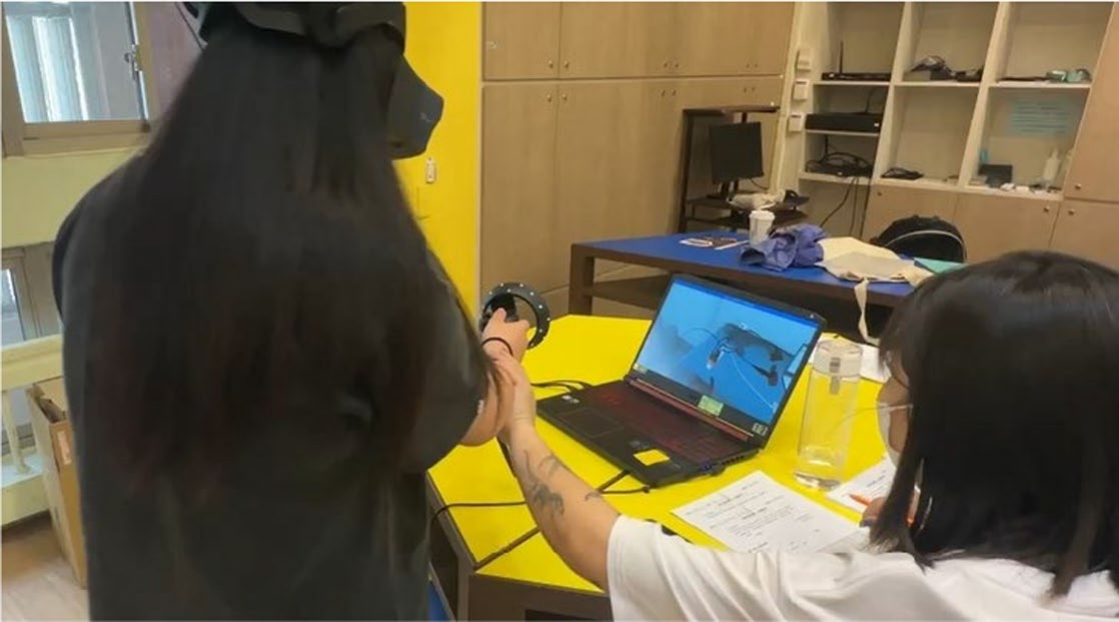
Facilitators providing support to students during the IVR-Warehouse experience.

At the beginning of each session, trained facilitators introduced the activity, demonstrated how to wear the headset, and explained the main controller functions, including pointing, selecting, confirming, and interacting with virtual objects. Students then completed a short practice session to familiarize themselves with the headset and controllers, reducing possible bias caused by unfamiliarity with VR operation. A brief pretest was administered to assess baseline warehouse safety knowledge. The pretest and posttest covered the same knowledge categories: Working at Heights, Electrical Safety, and Safe Cargo Handling. Students were divided into four groups per class and rotated to use the available VR headsets. Each participant completed the three IVR-Warehouse modules within approximately 3 to 5 min. During the modules, students identified unsafe conditions, selected hazards, and completed corrective actions in the virtual environment, such as addressing overloaded plugs, unsafe cargo stacking, and high-altitude work risks. Thus, the activity involved both decision-making and controller-based object interaction, rather than only quiz answering. During formal training, facilitators provided only technical and physical safety support, such as headset adjustment, collision prevention, and device troubleshooting. They did not provide answers or guide students’ safety-related decisions. After the IVR training, students completed the posttest and questionnaire measuring task-specific IVR self-efficacy, ECL, and OCL (Figure 4).

### 2.3 Participants

This study was conducted in the Commercial Management Department of a technical high school in Taiwan. A total of 183 Grade 10 students participated, and 167 valid responses were retained for data analysis, resulting in a valid response rate of 91.3%. Among the valid participants, 67 were male (40.1%) and 100 were female (59.9%), with a mean age of 17 years (SD = 0.51). A chi-square goodness-of-fit test showed that the gender distribution significantly differed from an equal distribution, χ^2^ (1, N = 167) = 6.52, *p* = 0.011. Prior VR experience was also recorded: 94 students (56.3%) had used VR before, whereas 73 students (43.7%) had no prior VR experience. An *a priori* power analysis using G*Power indicated that at least 77 participants were required for a medium effect size (f^2^ = 0.15), *α* = 0.05, power = 0.80, and three predictors; therefore, the final sample size was adequate. Participants were not randomly assigned because intact classes were used, and all students received the same IVR-Warehouse training. School and parental permission were obtained, and ethical approval was granted by the National Taiwan Normal University Ethics Committee (202412HS003).

### 2.4 Questionnaire

The task-specific IVR self-efficacy scale was adapted from Tai and Hong (2024) and included six initial items assessing learners’ confidence in operating the IVR-Warehouse system and completing warehouse-based IVR training tasks. The cognitive-load-related items were adapted from Zhang et al. (2025) based on Cognitive Load Theory (CLT) (Sweller, 2020). Items related to system response speed, fear of collision, loss of balance, and object manipulation difficulty were classified as extraneous cognitive load (ECL), whereas items related to attention maintenance, interface accessibility, controller difficulty, and action sequencing were classified as operational cognitive load (OCL). Although NASA-TLX is widely used to assess subjective workload, it was not adopted because it measures overall workload across multiple domains, including mental, physical, and temporal demands (Hart and Staveland, 1988). In contrast, this study required a CLT-aligned measure that could more specifically examine cognitive load in an IVR learning environment. To ensure content validity, clarity, and linguistic appropriateness, the Mandarin questionnaire was reviewed by five domain experts. In addition, five students reviewed the questionnaire items to assess their comprehensibility. The retained items were examined through CFA and reliability analysis. All items used a 5-point Likert scale from 1 (strongly disagree) to 5 (strongly agree).

### 2.5 Data analysis

Data analysis was conducted using SPSS 29 and AMOS 29. Descriptive statistics were first used to summarize participants’ demographic information, prior VR experience, questionnaire responses, and learning outcome. *A priori* power analysis was conducted using G*Power to examine sample adequacy. Chi-square analysis was used to examine whether prior VR experience differed by gender. The significance level was set at *α* = 0.05.

For the measurement analysis, confirmatory factor analysis (CFA) was conducted in AMOS 29 to examine construct validity. Items with factor loadings below 0.50 were removed. Item discrimination was examined using independent-samples *t* tests between the upper and lower 27% groups. Reliability and convergent validity were evaluated using Cronbach’s alpha, composite reliability (CR), and average variance extracted (AVE). Discriminant validity was examined by comparing the square root of AVE with inter-construct correlations.

For the structural model, path analysis was conducted in AMOS 29 to examine the hypothesized relationships among task-specific IVR self-efficacy, ECL, OCL, and learning outcome. Model fit was evaluated using χ^2^/df, RMSEA, GFI, AGFI, NFI, TLI, CFI, IFI, RFI, PNFI, PCFI, and PGFI. Because the Hazard Awareness Test showed substantial non-normality, with skewness = −2.60 and kurtosis = 8.86, non-parametric bootstrapping with 5,000 resamples was applied. Direct and indirect effects were considered significant when their 95% bootstrap confidence intervals did not include zero. Effect sizes were calculated using Cohen’s *f*^2^ based on the corresponding R^2^ values, with *f*^2^ values of 0.02, 0.15, and 0.35 interpreted as small, medium, and large effects, respectively.

## 3 Results

### 3.1 Item analysis

To examine internal construct validity, a confirmatory factor analysis (CFA) was conducted using SPSS AMOS 29. Following Hair et al. (2019), items with factor loadings (FL) below 0.50 were excluded. All retained items demonstrated satisfactory FL exceeding 0.50. The first-order CFA yielded acceptable model fit indices across constructs. Specifically, *χ^2^*/*df* values ranged from 0.304 to 2.387, RSMEA values ranged from 0.001 to 0.091, GFI values ranged from 0.986 to 0.998, and AGFI values ranged from 0.928 to 0.991. These results met the recommended thresholds proposed by Byrne (2016): *χ^2^/df* < 5, RMSEA < 0.10, and GFI and AGFI > 0.80 (see Table 2).

**Table 2:** Model fit indices for first-order confirmatory factor analysis of each construct.

Construct	*χ^2^*	*df.*	*χ^2^/df.*	RMSEA	GFI	AGFI
Threshold	—	—	< 5	< 0.10	> 0.80	> 0.80
IVRSE	4.774	2	2.387	0.091	0.986	0.928
ECL	1.608	2	0.804	0.002	0.995	0.975
ICL	0.609	2	0.304	0.001	0.998	0.991

To assess external validity, item discrimination was examined by dividing participants into upper and lower 27% groups based on total scores, and conducting independent samples *t* tests. As suggested by Green and Salkind (2016), a *t*-value greater than 3 (*p* < 0.001) indicates significant discrimination. The *t*-values for all retained items ranged from 14.101 to 21.292, demonstrating strong external validity (see Table 3). Based on this criterion, the IVR self-efficacy (IVRSE) scale was refined from six to four items, the ECL scale from six to four items, and the OCL scale also from six to four items (see Table 3).

**Table 3:** Summary of item analysis.

Item content by construct	*M*	*SD*	FL	*t-*value
IVR self-efficacy (IVRSE)				
I am confident in my ability to operate the “IVR Warehouse” task with ease.	4.132	1.062	0.925	20.953***
I can quickly identify the skills needed to operate the “IVR Warehouse” task.	4.144	1.043	0.930	21.292***
I feel confident in mastering the operational skills required during the “IVR Warehouse” experience.	4.168	1.010	0.918	20.456***
I am able to handle unexpected situations calmly during the “IVR Warehouse” experience.	4.066	1.048	0.850	16.657***
Extraneous cognitive load (ECL)				
During the “IVR Warehouse” experience, the VR system’s response speed (too fast or too slow) made it difficult for me to concentrate.	2.126	1.115	0.850	14.101***
I felt frustrated because I was afraid of bumping into walls or obstacles during the experience.	2.150	1.123	0.865	14.256***
I felt disoriented and frustrated due to a loss of balance during the VR experience.	2.030	1.132	0.890	14.946***
I felt frustrated when objects did not move according to my intentions during the VR experience.	2.108	1.157	0.882	14.717***
Intrinsic cognitive load (ICL)				
I often found it difficult to stay focused while engaging in the “IVR Warehouse” VR experience.	2.180	1.116	0.876	15.871***
I felt frustrated when I encountered interface commands that were hard to access in the VR environment.	2.090	1.156	0.896	16.583***
I felt discouraged when the VR controller was hard to use.	2.066	1.178	0.942	18.348***
I had difficulty understanding the sequence of actions in the VR game, which often led to frustration.	2.024	1.146	0.837	14.477***

*p < 0.05; **p < 0.01; ***p < 0.001.

### 3.2 Reliability and validity analysis

Reliability of the measurement model was examined by calculating Cronbach’s alpha (*α*) for internal consistency and composite reliability (CR) for external consistency. Following Cheung et al. (2023), α and CR values above 0.70 are regarded as acceptable. In this study, Cronbach’s α values ranged from 0.927 to 0.948, and CR values likewise fell between 0.927 and 0.948, demonstrating strong reliability across all constructs.

Convergent validity was examined using average variance extracted (AVE), with all constructs exceeding the recommended threshold of 0.50. AVE values ranged from 0.760 to 0.822, indicating satisfactory convergent validity. Discriminant validity was also established, as the square root of each construct’s AVE was greater than the inter-construct correlations, meeting the standard criteria (see Table 4). Overall, the measurement model exhibited strong internal consistency, convergent validity, and discriminant validity, confirming its reliability and construct validity (Table 5).

**Table 4:** Construct reliability and convergent validity.

Constructs	*M*	*SD*	Cronbach’s α	CR	AVE
IVRSE	4.127	0.968	0.948	0.948	0.822
ECL	2.103	1.025	0.927	0.927	0.760
ICL	2.090	1.053	0.937	0.937	0.790

**Table 5:** Construct discriminant validity.

Constructs	1	2	3
IVRSE	(0.907)		
ECL	−0.532***	(0.872)	
ICL	−0.503***	0.572***	(0.889)

*p < 0.05; **p < 0.01; ***p < 0.001.

### 3.3 Learning outcome

The learning outcome test items were designed to evaluate learners’ ability to recognize potential hazards and determine the appropriate safety procedures within warehouse operational contexts. These 12 items (i.e., 4 items for each unit) targeted multiple domains of warehouse safety, including high-altitude work, equipment operation, cargo handling, and electrical safety. Table 6 presents sample questions administered after participants completed the *VR-Warehouse* simulation. Correct answers are indicated in bold to highlight the intended responses.

**Table 6:** Sample posttest questions evaluating students’ learning outcomes.

	
Exception scenarios for height-related safety measures	
In the following descriptions of warehouse work situations, which depicts an incorrect behavior?	Before performing high-altitude work, Red secures the safety rope to a sturdy structure.
	While transporting goods, in order to ensure that the forklift load does not collapse, Yellow stands on the forks to hold it steady.
	Blue notices that Green is working at height without wearing a safety helmet and promptly reminds her.
	Before leaving work, Purple properly returns items to their place, turns off electrical equipment, and tidies up the environment.
Incorrect practices in electrical equipment use	
When using electrical equipment, which of the following statements is incorrect?	If electrical equipment or wiring catches fire, non-conductive fire extinguishing equipment, such as carbon dioxide or dry powder extinguishers, should be used.
	If equipment malfunctions but can be repaired quickly, there is no need to hang a warning tag.
	When using an extension cord, avoid coiling the cable and be mindful not to overload it.
	If electrical machinery is operating and an abnormal condition is detected, it should be reported immediately; if time does not permit, the power should first be cut off.
Safe practices for warehouse material placement	
When arranging materials in a warehouse space, which of the following statements is correct?	To free up aisle space, stack materials as high as possible.
	Temporarily place idle empty boxes in the aisle and put them away later when there’s time.
	To prevent materials from collapsing, they should be placed against the wall.
	None of the above.

The VR-warehouse Hazard Awareness Test scores ranged from 4.5 to 10 (*Median* = 8.0, *Mode* = 9.2). The distribution was highly negatively skewed (skewness = −2.60) and leptokurtic (kurtosis = 8.86), indicating a strong clustering of scores near the upper limit and the presence of extreme values (see Table 7).

**Table 7:** Descriptive statistics for the VR Warehouse hazard awareness test.

Min.	Max.	Median	Mode	Skewness	Kurtosis
4.4	10	8.0	9.2	−2.60	8.86

### 3.4 Model fit analysis

The model fit of the proposed structural equation model was evaluated using multiple indices, following the criteria recommended by Byrne (2016). An adequate fit is indicated by a *χ^2^/df* ratio less than 5, an RMSEA less than 0.08, and GFI, AGFI, NFI, NNFI, CFI, IFI, and RFI values greater than 0.80. In addition, PNFI, PCFI, and PGFI values should exceed 0.50.

The results indicated a good model fit: *χ^2^/df* = 1.873; RMSEA = 0.073; GFI = 0.905; AGFI = 0.861; NFI = 0.943; NNFI = 0.965; CFI = 0.972; IFI = 0.972; RFI = 0.928; PNFI = 0.749; PCFI = 0.773; and PGFI = 0.617 (see Table 8). All values met the recommended thresholds, suggesting that the structural model was well-fitted to the observed data.

**Table 8:** Model fit analysis.

	Threshold	Result
Absolute fit indices		
χ^2^*/df*	< 5	1.873
RMSEA	< 0.08	0.073
GFI	> 0.80	0.905
AGFI	> 0.80	0.861
Incremental fit indices		
NFI	> 0.80	0.943
TLI/NNFI	> 0.80	0.965
CFI	> 0.80	0.972
IFI	> 0.80	0.972
RFI	> 0.80	0.928
Parsimony-adjusted indices		
PNFI	> 0.50	0.749
PCFI	> 0.50	0.773
PGFI	> 0.50	0.617

### 3.5 Path analysis

**Figure 5:**
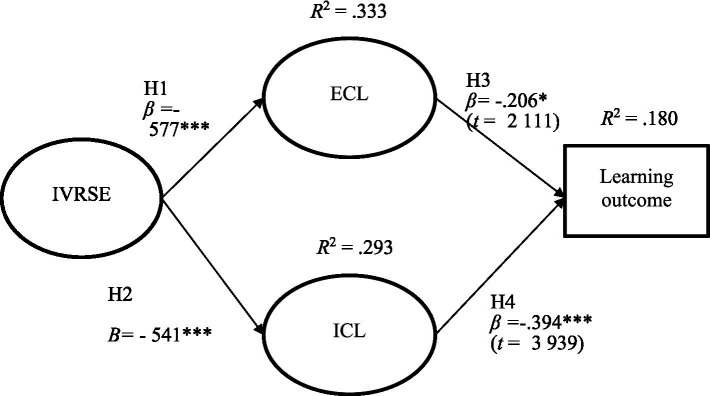
Path analysis. *p < 0.05; **p < 0.01; ***p < 0.001.

Path analysis using SPSS AMOS 29 showed that task-specific IVR self-efficacy (IVRSE) was negatively associated with both extraneous cognitive load (ECL) (*β* = −0.577, t = −7.593, *p* < 0.001) and VR operational cognitive load (OCL) (β = −0.541, t = −7.192, *p* < 0.001). Both forms of cognitive burden also negatively predicted learning outcome. Specifically, ECL had a significant negative effect on learning outcome (β = −0.206, t = −2.111, *p* < 0.05), whereas OCL showed a stronger negative effect (β = −0.394, t = −3.939, *p* < 0.001). Given the non-normal distribution of the Hazard Awareness Test, non-parametric bootstrapping with 5,000 resamples was applied to evaluate the robustness of the path estimates. The bootstrap confidence intervals for all direct effects did not include zero, supporting the statistical stability of the hypothesized paths. The indirect effect of task-specific IVRSE on learning outcome through ECL and OCL was also significant (β = 0.172, t = 2.879, *p* < 0.01), with a 95% bootstrap confidence interval [0.172, 0.426] that did not include zero. This result supported the hypothesized mediation effect. The full path results are presented in Table 9 and Figure 5.

**Table 9:** Summary of structural equation modeling.

Path analysis	Effect	SE	*t-*value	95% CI	
				LL	UL
Direct					
IVRSE → ECL	−0.577***	0.074	−7.593	−0.710	−0.430
IVRSE → ICL	−0.541***	0.075	−7.192	−0.687	−0.401
ECL → Learning performance	−0.206*	0.081	−2.111	−0.450	−0.047
ICL → Learning performance	−0.394***	0.078	−3.939	−0.447	−0.081
Indirect					
IVRSE → ECL & ICL → Learning performance	0.172**	0.077	2.879	0.172	0.426

*p < 0.05; **p < 0.01; ***p < 0.001.

## 4 Discussion

Virtual environments can replicate scenarios that are difficult, dangerous, or ethically problematic to stage in real life, while providing learners with an embodied sense of “being there” (McIntosh, 2022). Within the framework of social cognitive theory, self-efficacy is fundamental to supporting cognitive processes and promoting positive learning outcomes, particularly in IVR-based learning contexts (Ateş and Kölemen, 2025). Prior evidence further suggests that self-efficacy can strengthen learners’ psychological readiness and reduce perceived risks, thereby helping to lower cognitive burden during virtual learning experiences (Al-Kfairy et al., 2025).

In line with these theoretical perspectives, this study proposed and tested a research framework examining how task-specific IVR self-efficacy influences learners’ cognitive burden, specifically extraneous cognitive load (ECL) and VR operational cognitive load (OCL), as well as their learning outcome in IVR-Warehouse. The results supported the proposed hypotheses, showing that task-specific IVR self-efficacy was negatively associated with both ECL and OCL. In addition, both forms of cognitive burden negatively predicted learning outcome, with OCL showing a stronger negative effect than ECL. These findings indicate that learners who felt more capable of operating the IVR-Warehouse system experienced lower cognitive burden and achieved better learning outcome, whereas difficulties related to system operation, interface access, controller use, and action sequencing may have hindered students’ learning outcomes.

### 4.1 Hypothesis verification

Consistent with Cognitive Load Theory (CLT) and Social Cognitive Theory (SCT), this study found that task-specific IVR self-efficacy negatively predicted both extraneous cognitive load (ECL) and VR operational cognitive load (OCL), supporting Hypotheses 1 and 2. This finding is consistent with prior research showing that learners with higher self-efficacy tend to experience lower cognitive burden when completing complex, unfamiliar, or technology-mediated learning tasks (Jiang, 2023; Zhang et al., n.d.). Importantly, the role of ECL in this study should not be interpreted as evidence of poor graphic design. IVR-Warehouse provided visually detailed and contextually realistic warehouse scenarios; however, ECL may still arise from interaction-related demands, such as system response speed, fear of collision, disorientation, loss of balance, or difficulty manipulating virtual objects. Thus, the findings highlight the need to optimize controller usability, navigation support, and user orientation, rather than suggesting that the visual design was inadequate.

Hypotheses 3 and 4 were also supported, as both ECL and OCL negatively predicted learning outcome. These findings suggest that students’ learning may be hindered not only by design-induced distractions, such as inefficient information presentation or system-related interruptions, but also by operational burdens related to interface access, controller use, attention maintenance, and action sequencing. This result aligns with previous studies indicating that excessive cognitive demands can interfere with information processing, schema construction, and learning outcome in digital learning environments (Gkintoni et al., 2025; Paas and van Merriënboer, 2020).

Given the non-normal distribution of the learning outcome variable, the hypothesized effects were further examined using non-parametric bootstrapping. The bootstrap confidence intervals supported the stability of the direct effects and the overall indirect effect. Drawing on SCT, the results further indicated that task-specific IVR self-efficacy indirectly supported learning outcome by reducing ECL and OCL. This mediation effect supports prior work emphasizing the motivational and regulatory role of self-efficacy in immersive and technology-enhanced learning contexts (Ateş and Kölemen, 2025; Du et al., 2025). Overall, these findings support the integrated SCT–CLT framework proposed in this study and highlight the importance of learners’ task-specific confidence in managing cognitive demands during IVR-based warehouse safety training.

### 4.2 Response to research question

Excessive cognitive burden can hinder learning by overloading learners’ working memory and reducing the cognitive resources available for information processing and schema construction (Skulmowski, 2023). Although CLT provides a useful framework for explaining cognitive demands, its traditional load categories may require refinement in dynamic IVR environments, where learners must simultaneously process instructional content, operate controllers, navigate interfaces, and complete interactive task sequences (Gkintoni et al., 2025). In response to the research question, the findings showed that both extraneous cognitive load (ECL) and VR operational cognitive load (OCL) negatively predicted learning outcomes. Specifically, ECL had a significant negative effect on learning outcome (*β* = −0.206, *t* = −2.111, *p* < 0.05), while OCL showed a stronger negative effect (β = −0.394, *t* = −3.939, *p* < 0.001). This indicates that students’ learning in IVR-Warehouse was affected not only by system- or environment-related distractions, but also by the operational burden of maintaining attention, accessing interface commands, using controllers, and following action sequences. Thus, OCL appeared to be a particularly important barrier for novice learners in IVR-based warehouse safety training.

### 4.3 Implications

This study provides two key implications for the application of IVR in warehouse safety training. First, the context-specific IVR simulation, IVR-Warehouse, allowed learners to repeatedly engage with warehouse safety scenarios that reflected the Taiwanese logistics and warehouse training context. By interacting with scenario-based hazards and task-related actions, students were able to practice safety awareness in an immersive environment that would be difficult or risky to reproduce in real settings. Practically, IVR-Warehouse may serve as a useful training tool for students majoring in warehouse, logistics, or vocational safety-related fields, offering opportunities for individualized, repeated, and experiential practice. The findings also suggest that strengthening learners’ task-specific IVR self-efficacy may help reduce perceived cognitive burden during IVR-based training.

Second, the findings highlight the importance of distinguishing between design-related and operation-related sources of cognitive burden in IVR learning. In this study, ECL referred to unnecessary cognitive demands caused by system- or environment-related distractions, such as response speed, fear of collision, loss of balance, and object manipulation difficulty. In contrast, OCL referred to the operational burden associated with maintaining attention, accessing interface commands, using controllers, and following action sequences within the IVR environment. Although both ECL and OCL negatively predicted learning outcomes, OCL exerted a stronger effect. This suggests that, for novice learners, difficulties in operating the IVR system and completing action sequences may be more influential than general design-related distractions. Therefore, IVR training designers should simplify controller operations, improve interface accessibility, provide clear action guidance, and include sufficient practice before formal training to reduce operational cognitive burden and enhance learning effectiveness.

### 4.4 Limitations and future study

This study has several limitations. First, only three of the ten IVR-Warehouse modules were used due to time constraints. Although these modules represented important warehouse safety scenarios, future studies may include more modules to examine whether task-specific IVR self-efficacy, ECL, OCL, and learning outcomes remain consistent across different safety topics and task demands. Longer practice time or repeated exposure may also clarify whether learners’ cognitive burden decreases as they become more familiar with the IVR system, controllers, and action sequences.

Second, task-specific IVR self-efficacy was measured only after the IVR activity. Future research should assess learners’ confidence before and after training to examine whether IVR experience improves self-efficacy. Third, although facilitators provided only technical and safety support, their presence may still have influenced students’ cognitive load or engagement. Future studies should adopt a more standardized facilitator protocol to reduce possible intervention effects. Although presence was not measured in the present study, prior VR simulation research suggests that sense of presence may not always directly predict performance; instead, learners’ perception of the VR environment may be more closely associated with performance outcomes (Chevalier et al., 2025). Therefore, future research should examine how perceived presence, environmental perception, ECL, and OCL jointly explain learning outcome in IVR-based warehouse safety training.

## 5 Conclusion

Warehouse safety training in Taiwan, as in many other countries, has traditionally relied on video tutorials. However, research on extending this approach to immersive virtual reality (IVR) remains scare, particularly with respect to its potential for improving efficiency and reducing workplace accidents. The findings of this study demonstrate that IVR is an effective training method, strengthening safety competencies and learning outcomes while removing real-world risks. Accordingly, IVR presents a robust solution for safety training, safeguarding workers from the inherent challenges of warehouse operations.

To ensure contextual relevance, IVR training systems should be customized to match the specific characteristics of different workplace environments, thereby enhancing learners’ situational awareness and perceived sense of risk. Importantly, this study also revealed that VR operational cognitive load (OCL) and extraneous cognitive load (ECL) both significantly influence learning outcomes, although with differing levels of predictive power. These findings contribute to a deeper understanding of cognitive load in IVR-based training, and highlight the importance of tailoring IVR design not only for warehouse safety but also for broader applications in technology-enhanced learning environments.

## Data Availability

The original contributions presented in the study are included in the article/supplementary material, further inquiries can be directed to the corresponding author.
